# Selection of cortical dynamics for motor behaviour by the basal ganglia

**DOI:** 10.1007/s00422-015-0662-6

**Published:** 2015-11-04

**Authors:** Francesco Mannella, Gianluca Baldassarre

**Affiliations:** Laboratory of Computational Embodied Neuroscience, Institute of Cognitive Sciences and Technologies, National Research Council (CNR-ISTC-LOCEN), Via San Martino della Battaglia 44, 00185 Rome, Italy

**Keywords:** Basal ganglia, Cortex, Motor action, Selection, Cortical dynamics, Reservoir computing

## Abstract

The basal ganglia and cortex are strongly implicated in the control of motor preparation and execution. Re-entrant loops between these two brain areas are thought to determine the selection of motor repertoires for instrumental action. The nature of neural encoding and processing in the motor cortex as well as the way in which selection by the basal ganglia acts on them is currently debated. The classic view of the motor cortex implementing a direct mapping of information from perception to muscular responses is challenged by proposals viewing it as a set of dynamical systems controlling muscles. Consequently, the common idea that a competition between relatively segregated cortico-striato-nigro-thalamo-cortical channels selects patterns of activity in the motor cortex is no more sufficient to explain how action selection works. Here, we contribute to develop the dynamical view of the basal ganglia–cortical system by proposing a computational model in which a thalamo-cortical dynamical neural reservoir is modulated by disinhibitory selection of the basal ganglia guided by top-down information, so that it responds with different dynamics to the same bottom-up input. The model shows how different motor trajectories can so be produced by controlling the same set of joint actuators. Furthermore, the model shows how the basal ganglia might modulate cortical dynamics by preserving coarse-grained spatiotemporal information throughout cortico-cortical pathways.

**Electronic supplementary material:**

The online version of this article (doi:10.1007/s00422-015-0662-6) contains supplementary material, which is available to authorized users.

## Introduction

Preparation and execution of intentional movements requires the activity of the motor cortex. This cortical region forms re-entrant parallel loops with both the dorsolateral basal ganglia and the cerebellum (Middleton and Strick 2000; Caligiore et al. 2013). In particular, the interaction between the motor cortex and the basal ganglia seems to be organized in relatively segregated cortico-striato-nigro-thalamo-cortical (CSNTC) loops (Alexander et al. 1986; Haber 2003; Romanelli et al. 2005). Various computational approaches have been attempted to explain these loops as implementing motor sequence processing (Beiser and Houk 1998; Berns and Sejnowski 1998), or dimensionality reduction (Bar-Gad et al. 2003). One of the most accredited hypotheses to date is that they implement action selection (Mink 1996; Redgrave et al. 1999; Gurney et al. 2001).

There are two main issues in trying to explain how the motor basal ganglia–cortical loops work. A first issue concerns the nature of the neural encoding used by the motor cortex. This cortical region reaches both the brainstem motor centres and, more directly, the spinal motor neurons projecting to the muscles (Orlovsky et al. 1999; Ijspeert et al. 2007). Thus, the same cortical areas control muscles and subcortical motor centres encoding sophisticated motor patterns (Ijspeert 2008; Ciancio et al. 2013). Over the last two decades, various hypotheses about the representation of movements within the motor cortex have been proposed. A wide number of studies have interpreted data, mainly coming from single-cell electrophysiology, as a proof that the motor cortex implements a topological map of the body in which the activity of single cells can be directly related to the resulting forces acting on the muscles (Evarts 1968; Georgopoulos et al. 1982; Sergio et al. 2005). In this vein, several studies also related the behaviour of distinct motor populations to the control of parameters such as rotation, speed, or direction of movements (among others: Buys et al. 1996; Georgopoulos et al. 1986; Kakei et al. 1999; Wang et al. 2010). On the other hand, several findings indicate that individual neurons in the motor cortex directly project onto wide sets of muscles (Cheney and Fetz 1985), and that the activity of single cortical motor neurons is correlated with complex movements (grasping, reaching, climbing, chewing, etc.; Luppino and Rizzolatti 2000; Graziano and Aflalo 2007). These studies have opened up a new computational interpretation of the motor cortex as forming a set of dynamical systems with time variability and oscillations not directly encoding movement patterns (Churchland et al. 2010; Afshar et al. 2011; Churchland et al. 2012; Mattia et al. 2013).

A second issue, related to the nature of cortical encoding, regards the mechanisms through which the basal ganglia modulate cortical activity in order to select motor plans. A current view is that selection between different channels within CSNTC loops determines which cortical pattern or assembly of neurons will be dishinibited at the level of the cortico-thalamic motor loops (Redgrave et al. 1999; Gurney et al. 2001). In this view, each pattern encoded in an assembly of cortical neurons expresses a distinct motor programme. A channel can release from inhibition a distinct cortical pattern (among others Wickens et al. 1994; Graybiel 1998; Ponzi and Wickens 2010).^1^ This general idea of selection as a differential dishinibition of separated cortical modules has also been extended to explain the interaction between cortex and basal ganglia in cognitive tasks (for instance in the “Prefrontal cortex basal ganglia working memory” PBWM model by Frank et al. 2001; O’Reilly and Frank 2006). All these proposals, while focussing on the selection mechanisms, do not explain how the selected cortical assemblies control the execution of motor programs or cognitive processes.

Here we present a hypothesis reconciling the dynamical nature of cortical encoding with the idea that basal ganglia selection gates thalamo-cortical loops. We propose that selection does not (or not only) choose between different cortical assemblies, but rather between different *activity dynamics * within the same populations. More in detail, our proposal distinguishes two different processes. The first process consists in the selection of a distinct set of dynamics within a cortical module based on the accumulation of coarse-grained spatiotemporal information at the level of the basal ganglia. The second process regards the interaction between these cortical dynamics and those of other cortical and subcortical areas to gain top-down and bottom-up information. We will show a neural network model implementing this proposal. The model is formed by a dynamical reservoir reproducing the dynamics of a cortical module interacting with the selection mechanism within the basal ganglia which is implemented similarly to what done in Gurney et al. (2001). The model explains how a neural population in the motor cortex can be recruited to generate different movements with the same motor actuators. Furthermore, it shows how the proposed mechanisms can handle cyclic (rhythmic) and end-point (discrete) movements (e.g. a “scratching” movement or a “reaching” movement). In the following, Sect. 2 illustrates the model, in particular Sect. 2.1 illustrates how we used reservoir computing to model cortical dynamics, Sect. 2.2 gives a rationale of how selection processes in the basal ganglia are modelled here and Sect. 2.3 describes our computational hypothesis on how basal ganglia select different dynamics within cortex. Section 4 describes the neural architecture of a core module built to explain the computational hypothesis and a system-level architecture formed by two such core modules to explain the emerging properties of their interaction. Section 4 describes the implementation details of the core module as well as other further details in the implementation of the system-level architecture. Section 5 describes the behaviour of the core module used as the controller of a three-degree-of-freedom (DoF) 2D simulated kinematic arm and a 20-DoF simulated dynamic hand, in tasks requiring the selection and expression of different cyclic or end-point motor behaviours. This section also describes the behaviour of the system-level architecture controlling the same 2D three-DoF simulated kinematic arm in the same tasks. In this case lesions to different parts of the system were exploited for the analysis of its emerging properties.

## Selection of cortical dynamics

### Cortical reservoirs

Recently, various works have highlighted that reservoir computing can be a candidate computational approach to describe the nature of cortical encoding (Wang 2008; Rigotti et al. 2010; Dominey 2013; Hoerzer et al. 2014). In particular, reservoir networks fulfil two important requirements to model the cortex. First, they are complex distributed dynamical systems with the capacity of dealing with the time course of sensorimotor and cognitive processes. Second, they have a uniform microstructure, with internal connections randomly generated with parametrized procedures. Reservoir computing fundamental principles have been contextually introduced under the notions of *liquid state machines* (LSM; Maass et al. 2002) and *echo state networks* (ESN; Jaeger 2002). The idea was anticipated in a work by Dominey (1995) in which the author presented a computational model of cortical sensorimotor sequence learning to control the oculomotor system. LSM and ESM approaches mainly differ in the level of abstraction of the neural units they use. LSM models are usually composed of units that reproduce real neurons at the level of their spiking activity. ESN models are built on the basis of more abstract discrete or leaky-integrator sigmoidal units, leading to dynamical systems which are easier to analyze. We implemented the cortical module of the model presented here as an ESN (see Sect. 4.1). For an extensive review on reservoir computing, see Lukovsevivcius and Jaeger (2009).

**Fig. 1:**
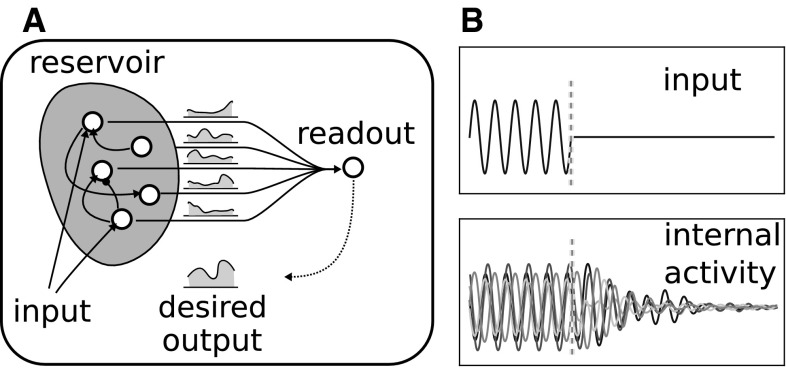
**a** General schema of the functioning of a dynamical reservoir. The units in the reservoir produce nonlinear dynamics which are temporal functions of the input signals. Weights to the read-out unit are modified to obtain a desired temporal function of the network activity. **b** An example of the internal dynamics of an echo state network: *on the top* a simple sinusoidal function as the input signal; *on the bottom* the resulting activities of a sample of units. It can be seen that the activity fades to zero after transient activity when the input signal is set to zero

Dynamical reservoirs are generally formed by a fully recurrent neural network with fixed, typically sparse, random weights, and one further layer of external units connected to the internal units to read out the dynamics of the network. The weights of the connections linking the reservoir units to the read-out units are suitably learned so that the temporal activity of the read-out units is a function of the internal activity of the reservoir. The weights of the internal connections of the reservoir are chosen so that the network activity has two features. First, the activity of its units fades to zero when there is no input and to a fixed-point attractor when there is a constant input (see Fig. 1b). This feature guarantees that the history of inputs is maintained in the activity of the network within a time window because input does not have indefinitely cumulative effects that would result in a chaotic behaviour of the network. As a consequence, if the interval between two inputs exceeds this temporal window, they will not interfere with each other in the modulation of the network activity. Second, the states of activation of the network units have a high variability during fading. This feature guaranties the richness of the temporal response of the reservoir to the input. As a result, the network can in principle reproduce any nonlinear temporal function with its read-out units. In other words, the more the states of the network are dissimilar from one another (low correlation between states in time), the more the time function that the network can learn and reproduce can be complex.

Thus in reservoir networks the internal encoding of input signals and the decoding of the internal activity to reproduce the output responses are two independent processes. Indeed, a reservoir is endowed with its own dynamics and encoding emerges spontaneously without any supervised teaching. This kind of network does not learn the features of the input signals but only converts them into a high-dimensional vector of nonlinearly changing neuronal activations resembling kernel methods used in support-vector networks (Cortes and Vapnik 1995), with the difference that the activations of the reservoir can be viewed as *temporal* kernels. Learning of a new decoding only updates the weights of the connections projecting from the units of the reservoir to the external read-out units (see Fig. 1a). After learning, a read-out unit transforms the temporal activity of the reservoir correspondent to an input sequence into a single nonlinear signal. Real cortical activity seems to share these features. Decoding of motor responses by reading the activity of motor cortex seems to be possible (Hatsopoulos et al. 2004; Golub et al. 2014). On the other hand, analysis on the same cortical activity reveals the presence of temporal dynamics that are not directly linked to motor responses (Churchland et al. 2012).

As an exception to this architecture, feedback from the external read-out to the internal units can also be present, e.g. to produce rhythmic behaviours without external input. Learning with feedback is not easy in reservoir networks, but various solutions have been found to implement it (Jaeger and Haas 2004; Steil 2004; Sussillo and Abbott 2009). In this case, the internal dynamics of the reservoir is able to acquire information about its output through the learning process. However, this information is mixed with the one coming from the input and transformed in the temporal dynamics of the network, so it could be isolated only with complex statistical methods.

### The basal ganglia

This section describes a way in which the basal ganglia might implement selection through disinhibitory competition. The role of the basal ganglia in action selection has been the subject of intense investigation, for example by Mink (1996), Redgrave et al. (1999) and Gurney et al. (2001) (see also Humphries and Gurney 2002; Gurney et al. 2004; Humphries et al. 2006; Bogacz and Gurney 2007). Gurney et al. (2001) proposed one of the most accredited computational hypothesis about the mechanisms behind basal ganglia selection. This hypothesis, together with the reservoir computing idea, is one of the key ingredients of our explanation about the interaction between the basal ganglia and cortex in the control of motor action. We now briefly describe the anatomical organization of the basal ganglia, and then our implementation of the selection hypothesis of Gurney et al. (2001) mentioned above.

**Fig. 2:**
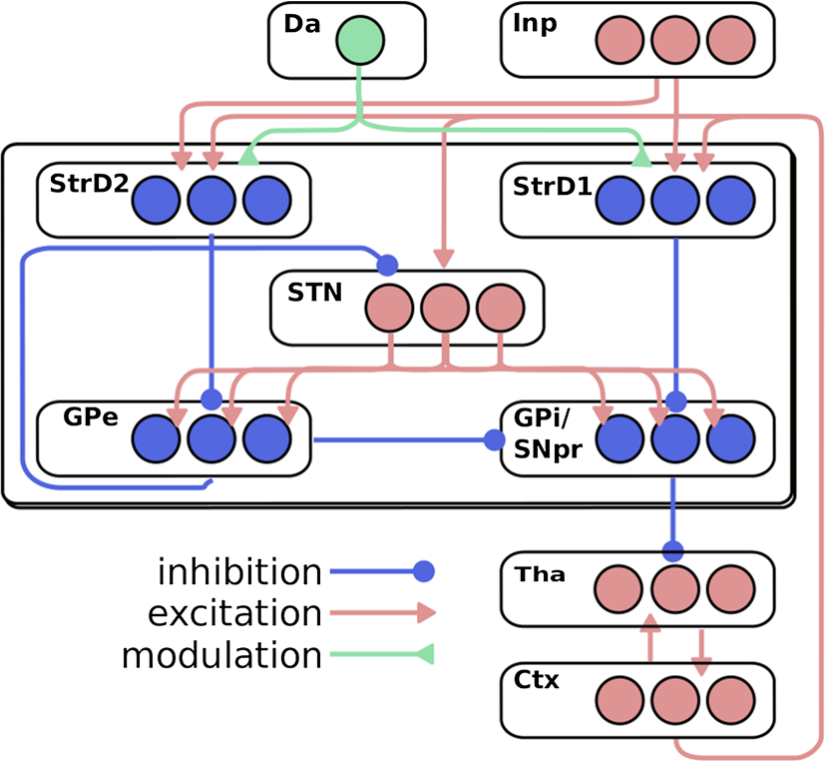
Schema of the intrinsic organization of the basal ganglia and their interaction with thalamic and cortical layers. *Arrows reaching the borders of the boxes* indicate that each unit of a sending layer reaches the corresponding unit of the target layer. In particular, each STN unit reaches all units of GPe and GPi. *Acronyms*: *Inp* input signal; *Da* dopamine efflux; *StrD*1 D1R-expressing striatal populations; *StrD*2 D2R-expressing striatal populations, *STN* subthalamic nucleus, *GPi* internal globus pallidus, *GPe* external globus pallidus, *Tha* thalamus, *Ctx* cortex

#### Intrinsic organization of the basal ganglia

Figure 2 shows the intrinsic organization of the basal ganglia and their interaction with the thalamo-cortical loops. The two main input projections of the basal ganglia come from the striatum (Str) and the subthalamic nucleus (STN). Both these nuclei receive most of their afferent projections from the cortex and send efferent projections to the GABAergic output nuclei of the basal ganglia, the internal globus pallidus (GPi) or the substantia nigra pars reticulata (SNpr). Str direct efferent projections to these regions originating from the medium spiny neurons form the *direct pathway*. These projections are GABAergic and reach subregions of the GPi/SNpr complex through parallel channels. STN efferent projections form the *hyper-direct pathway*. They are glutamatergic and spread diffusely over the GPi/SNpr output layers and the external globus pallidus (GPe). Projections from the Str to the GPe, and from there to the GPi/SNpr complex, form the *indirect pathway*. They are GABAergic and segregated in parallel substantially segregated channels similarly to those of the direct pathway. Str spiny neurons whose projections form the direct and indirect pathways are mainly distinguishable for two reasons. First, they tend to express two different families of dopamine receptors in different proportions. Neurons in the direct pathway tend to express more D1-like low-affinity dopamine receptors, while those in the indirect pathway tend to express more D2-like high-affinity dopamine receptors. Second, the direct pathway has a feed-forward organization. Instead, the indirect pathway consists in a multi-synaptic pathway involving a negative feedback circuit. Indeed, the GPe is reached by STN projections that are similar to those reaching the GPi/SNpr complex, with the difference that the former also sends back inhibitory projections to the STN itself (see Fig. 2). The organization in parallel segregated channels within the basal ganglia extends to the pathway going from the GPi/SNpr complex to the thalamus and then to the cortex which projects back to the Str and the STN. Along this pathway local populations maintain a relative segregation so that CSNTC parallel loops can be identified (Alexander et al. 1986; Parent and Hazrati 1995; Middleton and Strick 2000; Romanelli et al. 2005). Importantly, while there is wide evidence that striatal regions also receive information from cortical territories other than those within the same loop, there is instead little if no evidence of such “diagonal” (out of loop) afferent projections to the STN (Romanelli et al. 2005; Mathai and Smith 2011).

#### Selection within the basal ganglia

Gurney et al. (2001) show how the interaction between the direct and the hyper-direct projections leads to the emergence of centre-off fields of pallidal activations. In particular, a GPi–SNpr neural population reached by highly activated Str afferents is overall inhibited, while its neighbouring populations are excited by the STN glutamatergic projections. As a result, activations of different Str regions compete for the inhibition of the corresponding regions in the output layers through STN lateral excitation. Low differences in the activity of two competing Str regions produce higher differences in the inhibition of the tonic activity of the corresponding SNpr and GPi layers. This leads to the selective disinhibition of distinct thalamo-cortical loops. Moreover, cortical feedback projections to the Str and STN make the internal competition between channels a cumulative dynamical process, similar of those described in neural-field modelling (Si 1977; Erlhagen and Schoner 2002), with the difference that competition within these CSNTC channels is based on disinhibition rather than excitation (Bogacz and Gurney 2007).

#### Selection locking and unlocking

While the direct pathway and its interaction with the hyper-direct pathway implements the cumulative disinhibition described above, the indirect pathway has been proposed to control the activity passing through the direct/hyperdirect pathway (Gurney et al. 2001). In particular, in this view a lack of tonic dopamine enhances the activity of striatal spiny neurons projecting to the indirect pathway. This condition reduces the efficiency and persistence of basal ganglia selection by reducing the signal/noise ratio, so that the system can be released from a previous selection (see Sect. 5.1 for a detailed description of the process).

### Integrating cortex and basal ganglia: the key computational hypothesis

In this section, we discuss how the basal ganglia dishinibitory mechanism can act on a single cortical reservoir to select a specific dynamics within it. Cortical networks can be compared to dynamical reservoirs due to both their uniform microstructure and the temporal dynamics of their neural activity (Sect. 2.1). Our hypothesis is that such cortical reservoirs can be internally modulated by a selection mechanism similar to the one described in Sect. 2.2. Selection by the basal ganglia defines which kind of dynamical system a given cortical module instantiates by changing the modality of response of a part of it so that the cortical module becomes a specific function of the input signals.

This hypothesis requires two sets of assumptions. At the functional level, we assume that *direct cortico-cortical projections* and *selection by the basal ganglia* guide the cortical activity in two distinct ways. On the one side, *direct cortico-cortical projections* regulate cortical dynamics by transmitting fine-grained information that defines the step-by-step time course of the response of the target cortical modules. On the other side, *selection by the basal ganglia* modulates cortical activity at lower timescales. This feature emerges from two aspects of the selection mechanism. First, basal ganglia integrate in time the differences between different sources of information, filtering fluctuations that occur at fine timscales. Furthermore, once selection locks in (see Sect. 2.2) it becomes less sensitive to further changes in the input signals reaching the striatum. As a result, during selection a part of the thalamo-cortical loop is persistently released, and the activity of the corresponding cortical sub-population enhanced.

**Fig. 3:**
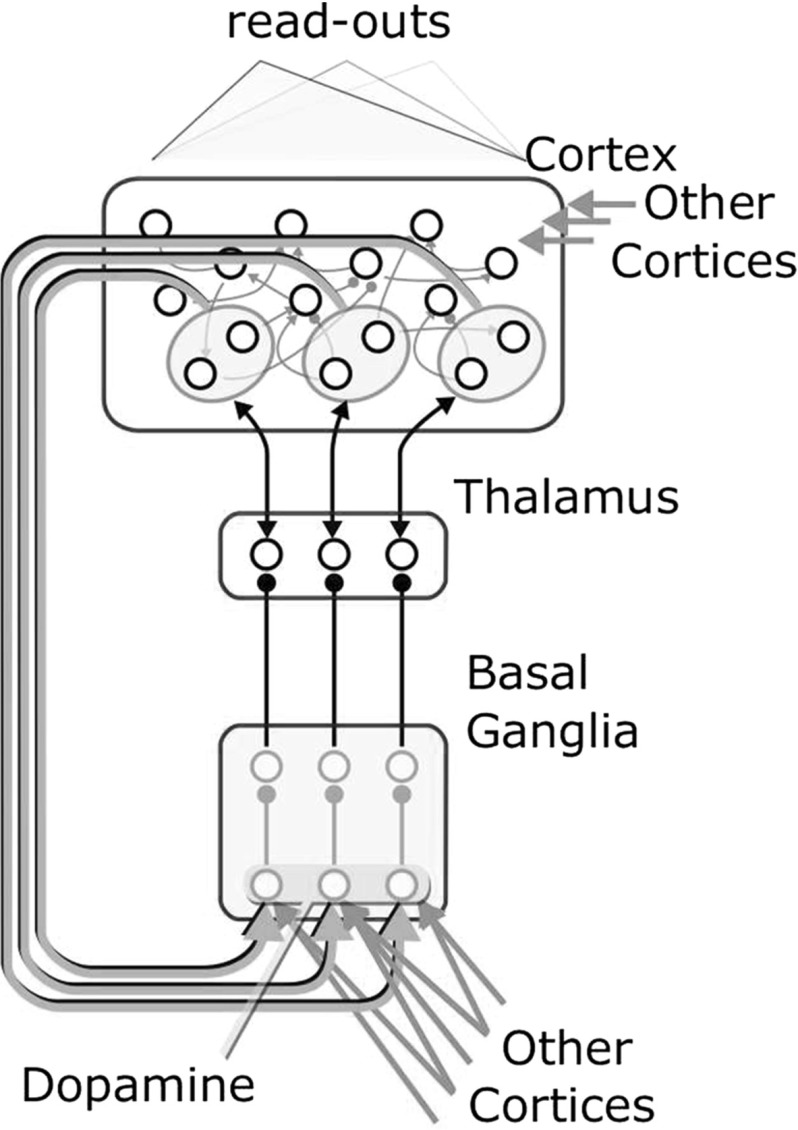
Organization of the interactions between a cortical module and the basal ganglia. Each channel within the basal ganglia projects to a sub-population of the thalamo-cortical loop. The cortical part of the sub-population projects back to the striatal input of the channel. Projections from other cortices to the striatum bias the differential activation of the channels. Direct projections to the cortical layer fuel its internal dynamics. The details of the intrinsic organization of the basal ganglia module are skipped in the picture (see Fig. 2)

At the structural level, we make three assumptions about the architecture of the basal ganglia-cortical system (see Fig. 3). First, different CSNTC channels reach a single cortical module. Second, those channels maintain themselves segregated within this module, reaching different subgroups of neurons. Third, all neurons within the cortical module maintain their uniformly sparse internal interconnectivity.

Afferent projections to this system reach two regions, the cortex and the input gates of the basal ganglia. According to the distinction made above, the two sets of projections have two distinct functional roles. While direct projections to the cortex feed the fine-scale dynamics of the reservoir, the projections to the basal ganglia feed the differential accumulation of evidence within the channels so as to bias selection on the basis of large-timescale information.

Simple static control signals (for instance steady excitatory signals coming from other cortices) would suffice for a reservoir network to be modulated in a similar way as done here by the basal ganglia (Sussillo and Abbott 2009). Why then should the cortex need a mechanism such as the competitive disinhibition implemented by the basal ganglia? The model presented here helps to highlight three possible answers. First, selection in the basal ganglia can be easily switched on and off based on neuromodulation. In particular, maintaining striatal dopamine at a high level keeps any selected channel steadily disinhibited. Instead, lowering dopaminergic efflux in the striatum releases the system thus allowing it to switch to another state (see Sect. 2.2). Parkinsonian patients in whom the efflux of dopamine to the striatum is impaired show several abnormalities in the voluntary initiation, speed and several other features of motor control (Muslimovic et al. 2007; Abbruzzese et al. 2009; Espay et al. 2011), thus revealing a main role of striatal dopamine in motor control. Simulation in Sect. 5.1 illustrates how selection in a CSNTC module is switched on/off by changing dopaminergic efflux to the striatum.

Second, maintaining static signals throughout cortico-cortical pathways is difficult. Indeed, following the computational assumptions we made about the nature of cortical dynamics (see Sect. 2.1) any information passing through a cortico-cortical pathway is temporally filtered, resulting in a complex nonlinear transformation. In the case of motor control, information about perception comes quite directly to the primary motor cortex from the somatosensory cortex. Instead, information about the overall movement to perform, originating from the environment and from internal states, reaches the motor cortex indirectly through the dorsal neural pathway, involving parietal and premotor cortex, and through the ventral pathway, involving the temporal, prefrontal and premotor cortex (Baldassarre et al. 2013). As a result, any top-down signal about the overall movement to perform depends on the dynamics of other cortical regions and would not be enough stable to serve for a steady selection of the internal dynamics. The same information filtered by a mechanism as the one of basal ganglia allows the production of steady signals that are robust to fine timescale perturbations. Furthermore, disinhibiting thalamo-cortical loops is a less interfering modulation on cortical activity than direct excitation. In particular, simulations described in Sect. 5 show that while extra excitation of a cortical area tends to saturate its activation, and thus to disrupt the information traversing it, its disinhibition leaves such information intact.

Third, learning task-relevant information at the level of the cortico-striatal synapses is simpler and faster than learning it at the level of cortico-cortical connections. In particular, with respect to the cortex, the basal ganglia can more easily perform the dimensionality reduction to isolate the coarse-grained categories relevant to decide which movement to perform (simulations in Sect. 5 will show this). How does this reconciles with the evidence of cortico-cortical plasticity (Buonomano and Merzenich 1998; Barth 2002; Fu and Zuo 2011) that might lead to learn the categories needed to select movements/tasks? Our idea is that learning at the striatal level occurs relatively fast, and so it can progressively guide the slower learning between cortical modules (Ashby et al. 2007; Shine and Shine 2014; Turner and Desmurget 2010). Following this idea, striatal inputs, once categorized, can steadily bias the selection of the dynamics of the target cortical module. This selection results in a better distinction between cortical dynamics which is easier to detect by learning processes operating at the level of cortico-cortical connections.

## Overview of the models

This section describes a neural architecture implementing the hypothesis described in Sect. 2, and a system-level model to study the interactions between multiple instances of such architecture. The description presented here is sufficient to understand the results, while all computational details of the implementation of these models are presented in Sect. 4. The first model (LOOP_MODEL) is composed of a CSNTC loop between a basal ganglia component and a cortical component (see Sect. 4). From now on, we will call this unit a *CSNTC module*. The architecture of a CSNTC module is shown in Fig. 3. The basal ganglia component is an implementation of the model of Gurney et al. (2001), consisting of 3 channels in loop with three different subpopulations of the cortical component (as in Fig. 2). Each of the three sub-populations is also in loop with a unit representing a thalamic population. Dopamine modulates the input to the units of the striatum in the basal ganglia component. Learning involves only the connections to the cortical read-out units. We used LOOP_MODEL to show that this hypothesized neural organization is able to select, based on the striatal dopaminergic efflux, differential dynamics given the same sensory contextual information and a differential information about the task. In particular, LOOP_MODEL is meant to describe the interaction between the primary motor cortex and the dorsolateral basal ganglia in the control of three different motor behaviours. Sections 5.1 and 5.2 show how this architecture can be used to control both cyclic and end-point movements.

**Fig. 4:**
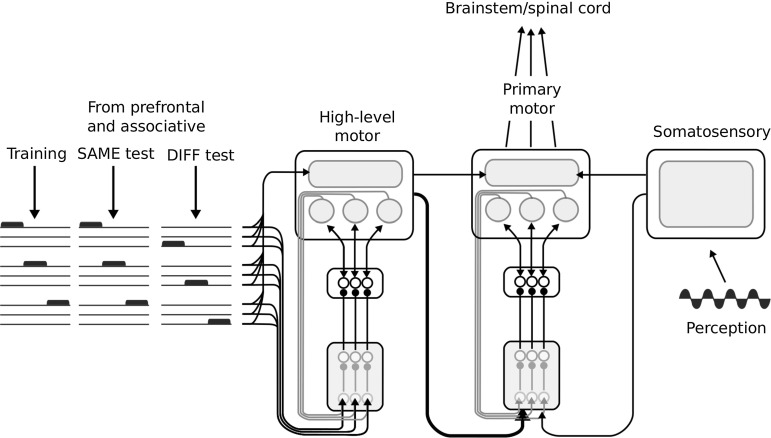
A system-level architecture describing the interaction between primary and higher-level basal ganglia–cortical loops. The model is formed by two CSNTC modules, the one *on the centre-left* representing an high-level motor area and the other *on the right* representing a primary motor area module. Sensory input comes from a cortical module representing the somatosensory cortex (*on the right*). *On the left* three examples of a train of higher-level input arrays abstracting information about the task coming from prefrontal and associative cortical areas. Each example contains three orthogonal binary input arrays defining three different tasks. Input arrays are grouped to form three categories encoding three different tasks in time. Such a categorization is hardwired in the connections to the high-level motor striatum. The connection in *red* reaching the primary motor striatum from the high-level motor cortex is the only cortico-striatal connection that is kept free to change, based on the learning rule described in Sect. 4.5 (colour figure online)

The second model (SYSTEM_MODEL, Fig. 4) is a system-level architecture explaining the interaction between multiple CSNTC modules. SYSTEM_MODEL is formed by two CSNTC modules and a further cortical module. In SYSTEM_MODEL one of the CSNTC modules (Fig. 4, *centre*) represents the primary motor loop and the read-out units of its cortical component directly control movements. The other CSNTC module (Fig. 4, *left*) represents a higher-level motor loop whose cortical output projects both to the striatum and to the cortex of the previous module. The cortical module at the right of Fig. 4 represents the somatosensory cortex. Its output reaches the striatum and the cortex of the primary motor module similarly to the high motor module (the somatosensory cortical module is not in loop with the basal ganglia, as it happens for primary sensory cortices). The SYSTEM_MODEL is directed to give a computational explanation of how the interaction between CSNTC modules allows for a better cortico-cortical communication of coarse-grained information, e.g. to control the different movements to perform. It also serves as a test of the role of cortico-striatal learning in defining how cortico-striatal information biases basal ganglia selection. A computational analysis of the possible ways to implement cortico-striatal learning is beyond the scope of this study. Instead, we introduce in Sect. 4.5 a simple unsupervised learning mechanism supporting category learning within the striatum (in future work, this mechanism might be strengthened with additional modulation of dopamine to implement reward-based learning). This unsupervised learning mechanism was implemented in the corticostriatal connections reaching the primary motor striatum from the high-level motor cortex. This simple learning mechanism is sufficient to illustrate our hypothesis on the role of the basal ganglia in filtering information at a coarse-grained spatiotemporal definition. Section 5.3 describes in a simulation the effect of lesioning these connections on the learning and expression of three motor tasks.

Throughout the paper we tested the model with three different tasks (hence basal ganglia have three channels) to simplify the visualization of the results. However, the model is able to scale to a higher number of tasks as shown in figure 1 in Online Resource 4.

## Computational details

This section illustrates the computational details of the various components of the model and the learning algorithms used to train the cortical read-out and the cortico-striatal connection weights.

### The cortical component

We implemented a cortical component as a reservoir network described by the following dynamical system:

1 $$\begin{aligned} \tau \dot{{\mathbf {u}}} = -{\mathbf {u}} + {\mathbf {W_{ux}}}{\mathbf {x}} + {\mathbf {W_{u}}}{\mathbf {z}} \end{aligned}$$

where ${\mathbf {u}}\in \mathbb {R}^N$ is the vector of activation potentials of the module units, ${\mathbf {x}}\in \mathbb {R}^M$ is the vector of external inputs to the component, ${\mathbf {W_{ux}}}\in \mathbb {R}^{M\times N}$ is the matrix of weights of the connections from the external inputs to the units of the reservoir, ${\mathbf {W_{u}}}\in \mathbb {R}^{N\times N}$ is the matrix of internal connection weights. ${\mathbf {z}}\in \mathbb {R}^N$ is the output function of the vector ${\mathbf {u}}$ given by:

2 $$\begin{aligned} {\mathbf {z}} = \left[ \tanh \left( \alpha \left( {\mathbf {u}}-th\right) \right) \right] ^{+} \end{aligned}$$

where $\alpha$ is the slope and *th* is the threshold of the function. This output function differs from the simple *tanh* function used in classical echo state models (Jaeger 2002) as it takes only its positive part. We preferred this transfer function so that the activation of the network units, viewed as activity of whole neural populations, has an higher biological plausibility (see Heiberg et al. 2013; Nordlie et al. 2010, for an analysis of rate models). The activity described in Eqs. 1 and 2 is used in all units in the models.

In all simulations lateral connection weights ${\mathbf {W_{u}}}$ were generated randomly and normalized following the constraints of leaky echo state reservoirs (Jaeger et al. 2007), with a further transfomation to improve the richness of the dynamics (see Appendix 1). In the case of online learning (see Sect. 4.4) read-out units are defined as in Eqs. 1 and 2 with the difference that the lateral connections between them are not present. In the case of offline learning (see Sect. 4.4) read-out units are just linear functions of inputs (as usually done in reservoir networks):

3 $$\begin{aligned} {\mathbf {o}} = {\mathbf {W}}_{oz}{\mathbf {z}} \end{aligned}$$

where $${\mathbf {o}} \in \mathbb {R}^O$$ and $${\mathbf {W}}_{oz} \in \mathbb {R}^{O\times N}$$.

Feedbacks from read-out units have been extensively studied among others by Jaeger and Haas (2004), Steil (2004), Sussillo and Abbott (2009), and Hoerzer et al. (2014). We chose not to explore this feature so as to maintain the simplicity of the models since the focus of this paper is on the system-level interaction between the basal ganglia and cortex.

### The basal ganglia component

The basal ganglia component was an implementation of the model of Gurney et al. (2001) with three channels (see Sect. 3). The model units were modelled through Eqs. 1 and 2. The microarchitecture of the module can be derived from Fig. 2. All layers were formed by three units. Each connection was a feedforward link between one unit and the topological corresponding unit in the following layer, thus reproducing in an abstract fashion the structure of basal ganglia partially segregated channels (one-to-one connections). The only exception to this was the STN, as each of its units was connected which all GPi and GPe units (all-to-all connections).

The modulation of dopaminergic efflux on the activity of striatal D1-expressing units was implemented as a *multiplicative excitatory* effect:

4 $$\begin{aligned} \tau \dot{{\mathbf {s}}}_{D1} = - {\mathbf {s}}_{D1} + (bl_{D1} + da_{D1})({\mathbf {W}}_{sc}{\mathbf {c}} + {\mathbf {W}}_{sx}{\mathbf {x}}) \end{aligned}$$

where ${\mathbf {s}}_{D1}$ is the vector of D1R-expressing striatal units, $bl_{D1}$ defines the responsiveness to the input not due to dopamine, $da_{D1}$ defines the responsiveness to the input depending on dopamine, ${\mathbf {c}}$ is the vector of inputs from the cortical units, ${\mathbf {W}}_{sc}$ is the matrix of weights of the connections between ${\mathbf {c}}$ and ${\mathbf {s}}$, ${\mathbf {x}}$ is the vector of activities reaching each channel from out-of-loop cortices, ${\mathbf {W}}_{sx}$ is the matrix of weights of the connections between ${\mathbf {x}}$ and ${\mathbf {s}}$.

The modulation of dopaminergic efflux on the activity of striatal D2-expressing units was implemented as a *multiplicative inhibitory* effect:

5 $$\begin{aligned} \tau \dot{{\mathbf {s}}}_{D2} = - {\mathbf {s}}_{D2} + \frac{1}{bl_{D2} + da_{D2}}({\mathbf {W}}_{sx}{\mathbf {c}} + {\mathbf {W}}_{xs}{\mathbf {x}}) \end{aligned}$$

where ${\mathbf {s}}_{D2}$ is the vector of D2R-expressing striatal units, $bl_{D2}$ defines the scale of responsiveness to the input not due to dopamine and $da_{D1}$ defines the scale of responsiveness to the input depending on dopamine (see also Fiore et al. 2014, for a similar implementation).

### The CSNTC module

The CSNTC module was implemented as a composition of a cortical module (Sect. 4.1) and a basal ganglia module (Sect. 4.2), as depicted in Fig. 2. The units of the cortical module project to the Str and the STN layers of the basal ganglia module. Direct input reaches the cortical module as well as the basal ganglia module.

### Learning the read-out weights

For the update of the connection weights to the read-out units, we used either batch regression or online learning methods. Regression was used to search the weights when computational speed was needed. Online learning was used to show that the target tasks could also be acquired in a biologically plausible way.

#### The batch method

For batch regression, we used Tikhonov regularization (Vogel 2002) as usually done in echo state networks optimization (Lukovsevivcius and Jaeger 2009). In particular we considered

- The training dataset ${\mathbf {Y}}= \left[ {\mathbf {Y}}_1 \dots {\mathbf {Y}}_i \dots {\mathbf {Y}}_Q \right] ^T$ where ${\mathbf {Y}}_i=\left[ {\mathbf {y}}_1 \dots {\mathbf {y}}_t \dots {\mathbf {y}}_S \right]$ is the array of data for a single desired trajectory and ${\mathbf {y}}_t=\left[ y_1 \dots y_O \right] ^T$ is the point at time *t* of the desired trajectory ${\mathbf {Y}}_i$.
- The input dataset ${\mathbf {X}} = \left[ {\mathbf {X}}_{1} \dots {\mathbf {X}}_{i} \dots ,{\mathbf {X}}_{Q} \right] ^T$ where ${\mathbf {X}}_i =\left[ {\mathbf {z}}_1 \dots {\mathbf {z}}_t \dots {\mathbf {z}}_S\right]$ is the array of input data related to a single desired trajectory and ${\mathbf {z}}_t \in \mathbb {R}^N$ is the vector of input at time *t*.

On this basis, the learning rule is as follows:

6 $$\begin{aligned} {\mathbf {W}}_{oz} = \left( {\mathbf {X}}^T{\mathbf {X}} + {\lambda }^2{\mathbf {I}}\right) ^{-1}{\mathbf {X}}^T{\mathbf {Y}} \end{aligned}$$

where ${\mathbf {W}}_{oz}$ is the array of read-out weights (see Eq. 3), ${\mathbf {I}}$ is the identity matrix and ${\lambda }$ is the regularization parameter.

#### The online method

We used the “backpropagation–decorrelation” (BPDC) algorithm described by Steil (2004) (see also Steil 2007) as the online learning method. We chose it because it has a low computational complexity (O(n)). BPDC has been studied in reservoirs where the read-out units belong to the reservoir and project feedback connections to the other neurons of the network. In BPDC, a decorrelation factor and an error backpropagation factor contribute to the modification of the weights reaching the read-out units. Since we limit our model to feedforward read-out units we can use a simplified version of the BPDC rule:

7 $$\begin{aligned} \Delta {\mathbf {W}}_{{oz}\ t+1} = \frac{\eta }{\Delta t} {\mathbf {g}}_{t+1} {{\mathbf {d}}^T_t} \end{aligned}$$

where $\eta$ is the learning rate, ${\mathbf {d}}_t$ is the decorrelation factor:

8 $$\begin{aligned} {\mathbf {d}}_t = \frac{{\mathbf {z}}_t }{ {\mathbf {z}}^T_t {\mathbf {z}}_t +{\mathbf {x}}^T_t {\mathbf {x}}_t + \beta } \end{aligned}$$

where $\beta$ is a regularization factor, and the backpropagation factor ${\mathbf {g}}_{t+1}$ simplified to the finite difference of the errors is:

9 $$\begin{aligned} {\mathbf {g}}_{t+1} = \left( 1 - \Delta t\right) {\mathbf {e}}_{t} - {\mathbf {e}}_{t+1} \end{aligned}$$

where ${\mathbf {e}}_{t} = {\mathbf {o}}_t - {\mathbf {y}}_t$ is the error between the current activations of the read-out units ${\mathbf {o}}_t$ and the vector ${\mathbf {y}}_t$ of the desired activations. In the original rule, the finite difference of the errors ${\mathbf {g}}_{t+1}$ is weighted by a backpropagation term involving the derivatives of read-out activations. This term depends on the autoconnections of the read-out units (Steil 2004), and reduces to zero in case of the absence of such autoconnections as in our model.

### Learning the cortico-striatal weights

In the simulations implementing SYSTEM_MODEL learning of the cortico-striatal connections was also simulated (see Sects. 3 and 5). In these cases, we used the unsupervised Oja learning rule (Oja 1982) for the update:

10 $$\begin{aligned} \Delta {\mathbf {W_{sx}}}_{t+1} = {\eta }_{sx}\left( {\mathbf {s}}_t {\mathbf {c}}^T_t - \left( \left( {\mathbf {s}}_t\odot {\mathbf {s}}_t \right) {\mathbf {1}}^T\right) \odot {\mathbf {W_{sx}}}_t \right) \end{aligned}$$

where $\eta _{sx}$ is the learning rate, $\mathbf {W}_{\mathbf {sx}}$ is the matrix of weights from a cortical layer outside the CSNTC module to the striatal layer within the CSNTC module, ${\mathbf {s}}$ is the vector of activities of the target striatal units (as in Eq. 2) filtered by a k-winner-takes-all (kWTA) function (here $k = 1$), $\mathbf {x}$ is the vector of activities of the cortical units filtered by a k-winner-takes-all (kWTA) function (here $k = 30$), ${\mathbf {1}}\in \mathbb {R}^{N_c}$ is a vector of all ones with the same length of $\mathbf {x}$, and $\odot$ is the element-wise multiplication operator. During the phase of cortico-striatal learning, Gaussian noise $\mathcal {N}\left( \mu ,\sigma \right)$ was also added to the activation of the striatal units to produce a random perturbation to the selection.

### Software

All simulations were implemented in C++ with the use of the *Armadillo open-source C++* library for linear algebra (see Sanderson 2010). Simulations were run on a Linux Debian Wheezy operating system hosted on a Intel I7 PC. The *Matplotlib* Python library (Hunter 2007) was used to produce plots and animations in all simulations with the three-DoF two-dimensional arm. The 20-DOF hand in the second set of simulations was implemented with the open-source *CENSLIB* library for 3-D scientific simulations (Mannella 2013) based on the *Bullet physics engine* (Coumans 2013). Data in the third set of simulations were analysed using the *R statistics and graphics* program (R Development Core Team 2008).

## Simulations

This section illustrates three sets of simulations of the models described in Sect. 3. The first set of simulations using LOOP_MODEL (Sect. 3) showed that the hypothesized neural organization is able to select, based on the striatal dopaminergic efflux, different dynamics and hence different rhythmic movements given the same sensory contextual information to cortex and a different information about the task to the basal ganglia. The second set of simulations involving LOOP_MODEL showed that the same model could also learn and produce fixed-point movements. Finally, a third set of simulations involving SYSTEM_MODEL (Sect. 3) showed the differential role of basal ganglia and cortex in motor control.

### Simulating motor control with a single CSNTC module

The idea described in Sect. 2.3 was first tested by implementing LOOP_MODEL that controls the motor behaviour of a simulated arm. In particular, the aim of this simulation was to show that LOOP_MODEL can select different dynamics given the same sensory contextual information and a different information about the task.

**Fig. 5:**
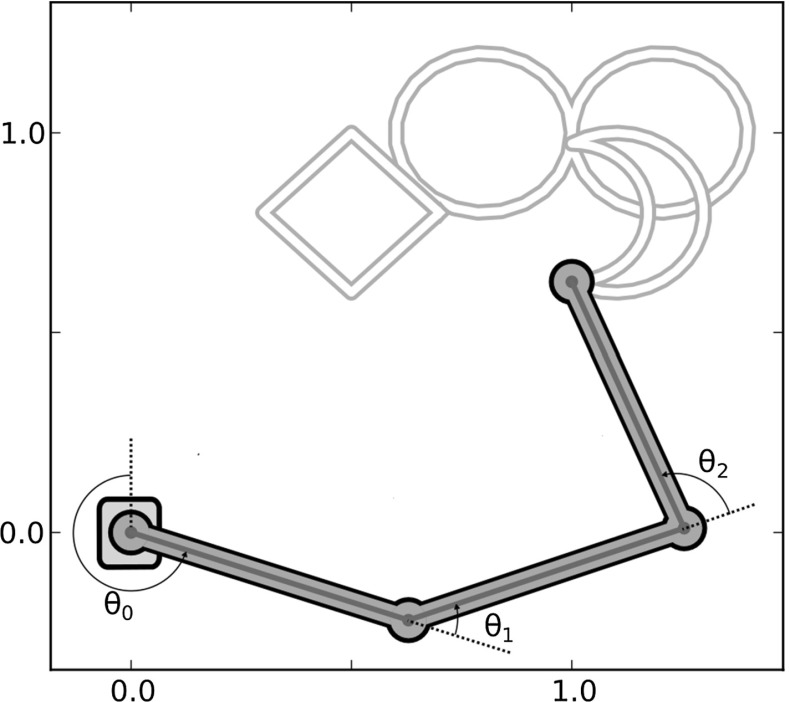
Schematic description of the two-dimensional kinematic arm used in the simulations. The three shapes *on the top* are the target trajectories to be learned. A *square*, a *sideways figure eight* and a *moon-like shape* can be recognized from the *top-centre* to the *top-right* of the figure

The simulation also showed how dopamine can play a key role in the on/off switching of the basal ganglia selection that leads to the learning of the target task. For simplicity we chose a two-dimensional simulated environment and a three-DoF articulated kinematic arm. Each of the three arm joints were controlled by a distinct read-out unit of the model. The task consisted in reproducing three different periodic behaviours that could be visually interpreted as writing a square, a sideways “8” shape and a moon-like shape (see Fig. 5). On the controller side, this corresponded to learning and reproducing three different sequences of read-out activities based on the selection of one of the three different basal ganglia channels (Fig. 3).

The simulation was subdivided in a learning phase and a test phase. Each phase was composed of several sessions. During a session each of the three behaviours was recalled once in random order, giving rise to three “trials.” During each trial a binary signal was sent to one of the striatal channels to bias selection. This binary signal represented information received by the basal ganglia component of the module from cortical or thalamic regions outside the module. A bottom-up context information, formed by a sinusoidal wave was directly sent to the cortical component of the module. This sinusoidal wave was the same throughout all trials in the simulation. It represented information directly coming to the cortical module from other cortical regions. Within each trial, the dopaminergic efflux was switched on after a short interval from the trial onset and was switched off before its end. We also defined a time window, which we called “task window,” internal to the dopaminergic efflux interval: learning took place within these task windows. This ensured that the cortical activity only depended on direct cortical input and basal ganglia disinhibition and not on perturbations due to the trial onset. Importantly, there was no reset between sessions, trials, or anywhere else throughout the whole simulation thus testing the capacity of the system dynamics to autonomously handle such transitions. In the initial training phase, the read-out weights were updated via an online learning or batch learning process, in distinct simulations (see Sect. 4.4 for details). The duration of the training phase depended on the kind of learning that was implemented. Online learning (see Sect. 7) consisted in 1000 sessions in which the read-out weights were updated in order to fit the desired trajectory. Batch learning required one session to store the array of cortical activations. Here for simplicity, we only describe the results obtained using the batch learning process (regression). The test phase was composed of three sessions. The first two sessions served to guaranty that the behaviour is stable after it is learned. The error (normalized root-mean-square error—NRMSE) was measured over the three task windows in the three trials, involving the three movements, of the last test session.

**Fig. 6:**
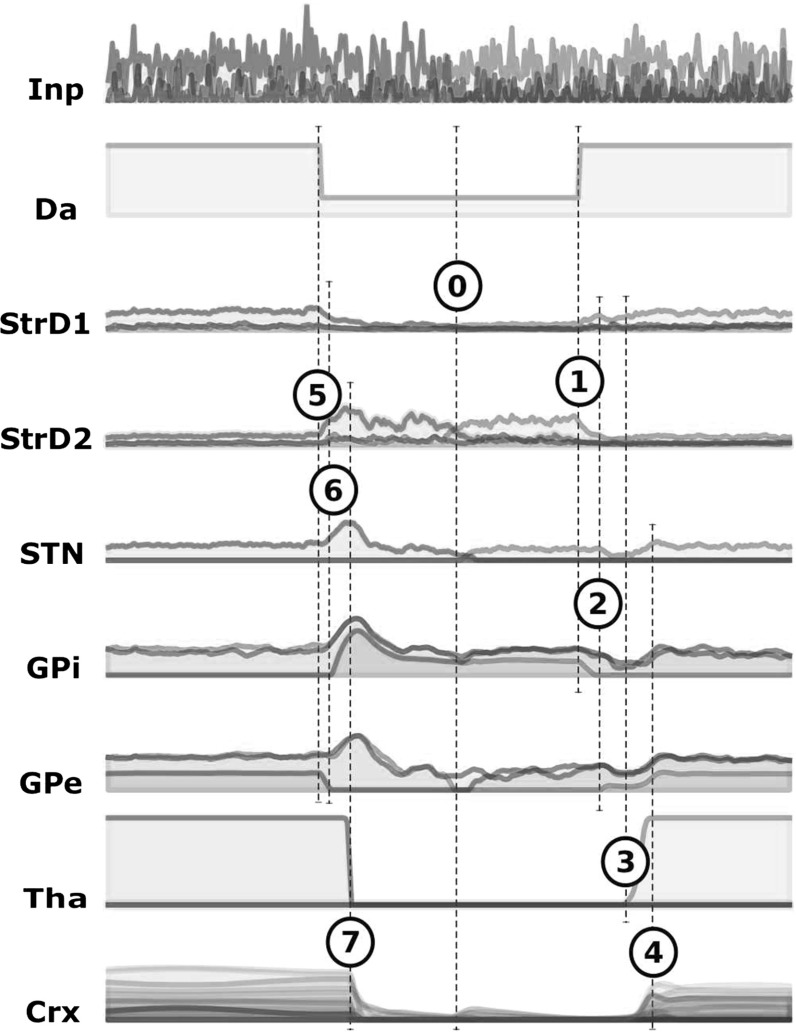
Simulations of the single CSNTC module architecture (LOOP_MODEL). Course of basal ganglia activity in a CSNTC module with three channels in the transition between the first and the second test trial: The *top row* shows the input signals reaching the three channels from other cortices (outside the CSNTC module) are shown. The input signal to the *green* channel is initially higher than the others. In the *middle* of the course of activity the input signal to the *red* channel becomes the highest. *0* *Da* activity is low. The network is in a low-energy state. Changing the input signals does not affect basal ganglia activity. *1* As soon as *Da* activity becomes high, activity in *StrD*1 grows while the corresponding activity *StrD*2 get steady low. *2* This change produces inhibition of the highly activated channel in the *GPi* layer. *3*, *4* The network reaches a new equilibrium where activity in the highly activated channel is in an up state throughout layers *StrD*1, *STN*, *Tha*, and *Ctx*. This equilibrium persists even when the input signal goes off and only a lowering of *Da* activity interrupts it. *5* Activations in *StrD*1 revert to a down state, while those of *StrD*2 become lower and with temporary peaks. *6* Differences between channels fade back to low values in the *GPi*. *7* *StrD*1, *STN*, *Tha*, and *Ctx* revert to down-state activity. *Acronyms* *Inp* input signal; *Da* dopamine efflux; *StrD*1 D1R-expressing striatal populations; *StrD*2 D2R-expressing striatal populations; *STN* subthalamic nucleus; *GPi* internal globus pallidus; *GPe* external globus pallidus; *Tha* thalamus; *Ctx* cortex (colour figure online)

**Fig. 7:**
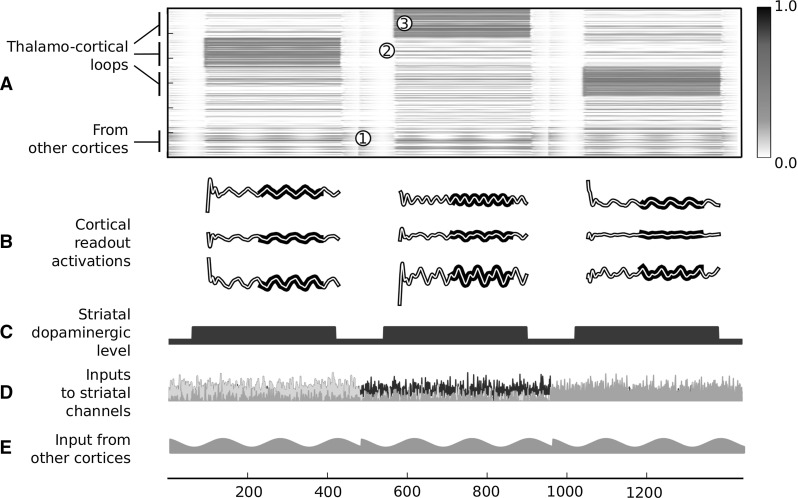
Simulations of the single CSNTC module architecture (LOOP_MODEL). Cortical activity during three trials of a test session. *a* Raster plot of the activity of the units in the cortical component. The *first half of rows on the top* shows the activity of the units connected in loop with the three thalamic channels. The *graph* clearly shows the switching from a down state to an up state of each subgroup of cortical units when the related thalamic loop is disinhibited. The last 20 % of rows on the bottom show the activation of the set of units that is reached by the cortico-cortical input (see *e*), whereas the remaining units are not reached by any input. *b* Activation of the three read-out units during the testing time window. The *bold black lines* stress the target output that had to be learned. Their duration denotes the learning time window. *c* Striatal dopaminergic efflux. Dopamine is set at a high level during each trial and at a low level between trials. *d* Cortical input to the three channels of the striatum. Gaussian noise is added to each signal. *e* Sinusoidal input reaching a set of units of the cortical module

Figure 6 shows basal ganglia activity in the test phase, focusing on the transition between the end of a trial and the beginning of the following one. This transition can be described in relation to the dopaminergic concentration in the striatum:

- *High dopamine*: If the concentration of dopamine at the striatal synapses moves from a low level to a high level, the activity of all D2R-expressing Str populations stabilizes at low values, while the activity of the selected D1R-expressing Str population starts growing (Fig. 6, point 1). This change produces a selective inhibition of the highly activated channel in the GPi layer, while a similar selective inhibition is removed in the GPe layer (Fig. 6, point 2). The overall increase in GPe activity produces a temporary deactivation of the STN layer. As a consequence, the overall activity of GPi is lowered allowing the dishinibition of a thalamo-cortical loop. *Lock-in*: As long as disinhibition of the thalamo-cortical loop persists, cortical increased activation excites the Str and the STN (see Fig. 6, point 3). Str, STN and cortical neurons belonging to the selected channel switch to an up state of activation, in a feedback loop reaction, and selection becomes locked-in (see Fig. 6, point 4).
- *Low dopamine*: If the concentration of dopamine at the striatal synapses moves from a high level to a low level, the D2R-expressing population is free to react to inputs and to inhibit the GPe (see Fig. 6, point 5). This activity breaks the equilibrium within the GPe-STN loop (see Fig. 6, point 6). *Unlock*: The level of activity of SNpr-GPi neurons cannot be reliably maintained below threshold anymore, the thalamus becomes inhibited, and cortical activity turns back to a down state, thus unlocking the network (see Fig. 6, point 7).

Within the model, all these dynamical events require a background activity in the cortical layer to happen. Without this, there is no thalamic activity, and thus the recurrent activity within the loops is null.

**Fig. 8:**
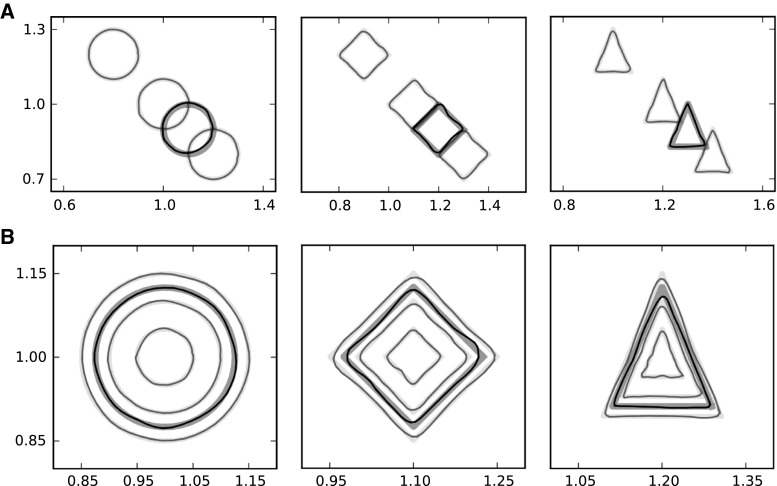
Simulations of the single CSNTC module architecture (LOOP_MODEL) showing the model capacity of generalization over scaling and translation. *Each column of graphs* shows the behaviour of the controlled 2D arm in case of the selection of one of the three basal ganglia channels. *Bold light grey curves* denote the target trajectories. *Bold dark grey curves* denote the trajectories expected during the generalization tests. The *thinner curves* show the trajectories actually performed in the three target tests and in the generalization tests. The *top row of graphs* **a** shows the case in which the same trajectory has been learned at three different spatial positions. The *bottom row* **b** shows the case in which the same trajectory has been learned at three different scales

Figure 7 shows cortical activity in the test phase of a typical simulation. During each trial, the following events take place:

1. Direct inputs to the cortex trigger the reservoir activity. Cortical activity stays at low levels if the selection process is not locked-in due to a lack of dopamine in the striatal layer (see Fig. 7a, point 1).
2. When dopamine efflux increases, inputs to the basal ganglia from other cortices bias the competition so that one of the channels is disinhibited (see Fig. 7a, points 2, 3).
3. Activity of the cortical neurons in loop with the disinhibited thalamic region is amplified (see Fig. 7a, point 3).

The presence of a highly activated neural population within the reservoir when a channel is locked-in has consequences on the whole cortical activity. As a result the cortical dynamics during the three task windows are different from each other, even though the sinusoidal signal activating the cortex is the same. Thus, when selection is steadily locked-in, the behaviour of the network is a well-determined temporal function of its inputs. Consequently, the weights to a read-out unit can be modified so that its activation follows a desired behaviour. Figure 7b shows the activity of the three read-out units in a test done after such a learning. It can be seen that the same read-out unit is capable of decoding the three dynamics of the cortical network into three distinct temporal patterns of activity. A video of the test phase of this simulation is given in Online Resource 1. Figure 1 in Online Resource 4 shows the behaviour of the model in the case in which it learns and reproduces four different motor trajectories instead of three to show how the model can learn a large number of patterns.

**Fig. 9:**
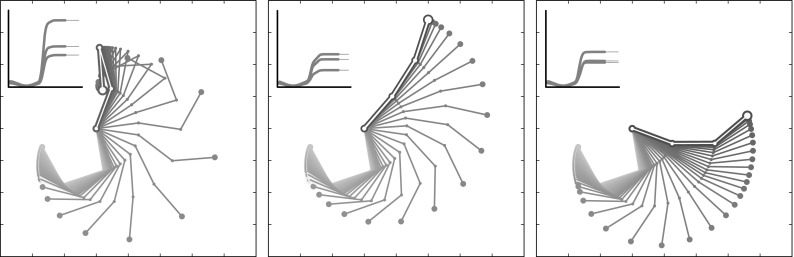
Simulations of the single CSNTC module architecture (LOOP_MODEL) showing the capacity of the model to learn and perform discrete movements. The *three graphs* show the trajectories of the arm while reaching each of the three the target postures (*white* and *red*). The *top-left of each graph* shows a plot of the modification in time of the angles of the three arm joints (colour figure online)

We also performed some tests to show how LOOP_MODEL is able to generalize learned motor trajectories over different features of the movement, for example scale or translation. In these tests, during the initial phase each target trajectory was learned in three different positions (see Fig. 8a, light grey curves) or at three different scales (see Fig. 8b, light grey curves). A further input signal was added to the sinusoidal signal going to the reservoir component of the model. This additional input signal was a constant signal whose amplitude varied based on the amount of translation or scaling of the trajectory. In the following test phase, a generalization test was added to the tests of the three learned trajectories. In this generalization test, the amplitude of the constant input signal did not correspond to any of the three amplitudes that were experienced during the learning phase but was rather a value between two of them. The results of these tests show that the model performs a motor trajectory that is translated or scaled in proportion to the level of the constant signal along a continuum that generalizes over the three learning samples (see Fig. 8a, b, dark grey curves). In this simulation, we used simpler shapes than before as target trajectories. This was done to clearly decouple the scaling factor from of the translation factor. Figure 2 in Online Resource 4 shows the same simulation with the previously described more complex trajectories.

### Simulating end-point motor control

A further simulation was implemented to show how LOOP_MODEL can learn not only rhythmic (limit-cycle attractor) movements but also discrete (fixed-point attractor) movements. All settings were as those of the first simulation with the only difference consisting in the use of three fixed-point target trajectories instead of the ones described in Fig. 5. Figure 9 shows the results of a typical test session. The three plots represent the movements of the arm to reach three different final targets. On the top-left of each plot a graph shows the activations of the read-out units in the three joints of the arm. It can be seen that, after learning, the model is able to produce the desired posture.

To test how the model scaled to real scenarios in motor control, we further tested it as a controller of a 20-DoF dynamic hand in a 3D physics simulator. The task was similar to the ones just described and consisted in moving the hand so to reach one of three different postures based on the task information. The model could easily learn all the three desired postures and reproduce them based on the related input. Learning of the read-out units was implemented via the online learning rule described in Sect. 4.4. A video of the simulation is given in Online Resource 2.

### Simulating the interaction between high-level and primary motor modules

The third set of simulations implemented SYSTEM_MODEL described in Sect. 2.3. The aim of this set of simulations was to illustrate how selection implemented by the basal ganglia maintains the task information throughout cortico-cortical pathways. Furthermore, it illustrates how a simple unsupervised cortical–striatal learning process is sufficient to allow the reduction of the dimensionality of cortical input to the striatum and extract information about the task (see Sect. 2.3). This architecture was tested, as the previous one, as a controller of a three-DoF kinematic arm acting in a two-dimensional simulated environment. Similarly to the previous case, each of the three joints of the arm were controlled by a distinct read-out unit of the model. Also the task was the same. Two types of information reached the controller. These two sources were intended to reproduce the difference between low-level sensory information and high-level task information. A first input carried the information about the trial task, that is about the trajectory to perform. This information represented the modulation by the prefrontal cortices, here abstracted as a binary vector signal (depicted on the left of Fig. 4). In particular, nine channels subdivided in three groups conveyed the signal about which of the three motor actions had to be carried out. Each channel in a group reached the same unit of the striatum of the high-level motor CSNTC module. Each channel also reached the cortical part of the same CSNTC module in a distributed way, with randomly chosen weights. The reason for this abstraction was that we were interested, as in the previous set of simulations, in reproducing the effect of a task-related high-level coarse-grained information on the selection of the cortical dynamics. A second input represented the information arriving to the somatosensory cortex from sensors. We abstracted this information as a sinusoidal signal (see the bottom-right of Fig. 4). This sinusoidal signal reached the somatosensory module in a distributed way, with randomly chosen connection weights. The reason for this abstraction was that, as above, we were interested, as in the previous set of simulations, in reproducing the effect of a sensory-related low-level fine-grained information on the maintenance of the cortical dynamics.

Learning happened at two levels. One learning process involved the connections going from the high-level motor cortical module to the striatum of the primary motor CSNTC module. A second learning process involved the connections going from the primary motor cortical module to its read-out units (the top-centre of the figure). Learning the connections between external input (from prefrontal cortex) and the high-level motor striatum was instead abstracted by implementing hardwired connections, as in this case unsupervised learning is not sufficient. Indeed, information about the desired categories to be acquired is not contained in the prefrontal information, and further motivational information would be needed. Thus in this case a reward-based learning process, the study of which was out of the scope of this work, should have been implemented.

**Fig. 10:**
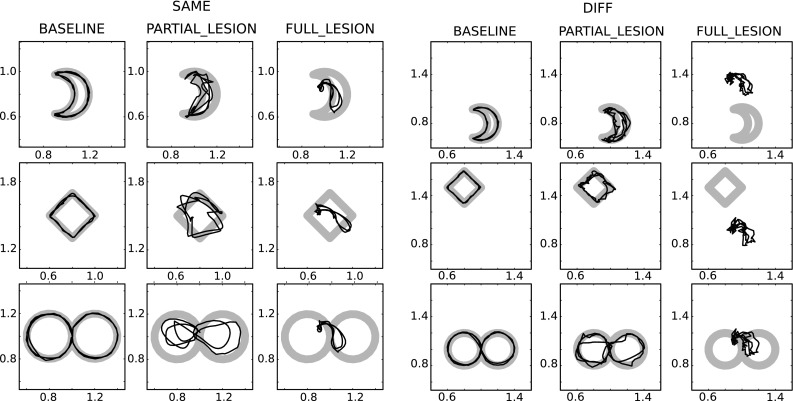
Simulations of the system-level architecture composed of two CNSTC modules (SYSTEM_MODEL). Performance of the model in the execution of the three tasks during the SAME test condition in each of the three kinds of simulations. The grid *on the left* shows the trajectories in the SAME test conditions, while the one *on the right* shows the trajectories in the DIFF test conditions. Within each grid the *left column* shows the performance in the BASELINE simulations. In both cases the trajectories of the arm follow the target with a very small error. Within each grid the *centre column* shows the performance in the PARTIAL_LESION simulations. In both cases, the error increases. All trajectories are centred on the target shapes. Within each grid the *right column* shows the performance in the FULL_LESION simulations. In both cases the shape of the trajectories is completely lost in the reproduction. In the SAME condition, the only information maintained is the position of the target shape in space

The simulation was divided in three phases:

- *First training phase* Cortico-striatal weights from the high-level motor cortical module to the primary motor striatum were updated using the unsupervised learning rule described in Sect. 4.5. During this phase, random noise was added to the striatal input of the primary motor module so that selection could happen randomly at the beginning. This phase lasted 30 sessions.
- *Second training phase* The read-out weights from the primary motor cortical module to the subcortical actuators were updated in a supervised manner, as described in Sect. 4.4. This phase lasted three sessions in the bach learning version, and 1000 sessions in the online version.
- *Test phase* The behaviour was tested in two different conditions. In one condition (SAME), the temporal pattern of binary vector signals reaching the higher-level motor module was the same as in the training phases. In the other condition (DIFF), the last bit within each group was switched on to encode the desired trajectory, instead of the first bit of the same group as done in the previous phases (see Fig. 4, on the left). Thus the binary vector signals in the two conditions were orthogonal, and their belonging to the same groups of information could be optimally detected only through reward-based clustering (here abstracted with hardwired connections) at the level of the striatum of the high-level CNSTC module.

Three simulations were preformed to show the function played by the different components of the model.

- BASELINE All connections in the architecture were intact. This represented the control condition of the experiment.
- PARTIAL_LESION The cortico-striatal connection between the high-level motor module and the primary motor module was lesioned before the learning processes. This condition tested the hypothesis for which the task information coming from the high-level motor module must be passed to the striatum of the primary motor module in order to optimize the selection of the right cortical dynamics.
- FULL_LESION Both the cortico-striatal connections between the prefrontal/associative input and the high-level motor module and the cortico-striatal connection between the high-level motor module and the primary motor module were lesioned before the learning processes. This condition tested the hypothesis for which in the model the task information is almost completely lost at the level of primary motor control when it is not filtered by the basal ganglia.

**Fig. 11:**
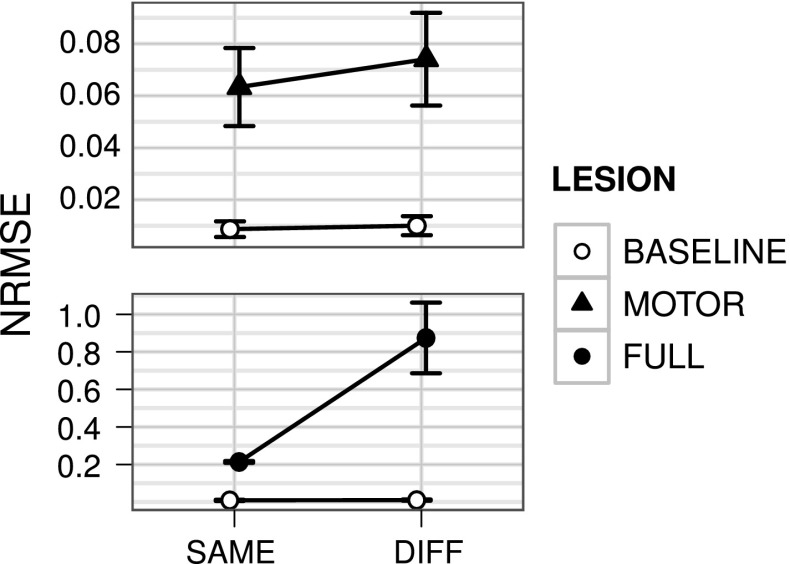
Simulations of the system-level architecture composed of two CNSTC modules (SYSTEM_MODEL). NRMSE means of BASELINE, MOTOR and FULL simulation groups. *Top* Means and standard errors of the BASELINE and MOTOR simulations are compared in the SAME and DIFF test conditions. *Bottom* Means and standard errors of the BASELINE and FULL simulations are compared similarly in the SAME and DIFF test conditions. Each set of simulations was composed of 100 simulations with different random number generator seeds. Note the different *y*-axis scale of the two graphs

Each kind of simulation was repeated 100 times, each time setting a different random number generator seed so that each simulation could be considered a test on a different individual.

In the case of the BASELINE simulation, when the binary vector signal about the task was the same as in the training phase (SAME condition), the resulting behaviour was a correct reproduction of the requested behaviour (Fig. 10, left column). Furthermore, in the DIFF test condition, the measured error (NRMSE) was only slightly higher than the one in the SAME condition (see Fig. 11).

In the case of the PARTIAL_LESION simulation, the resulting behaviour during the test phase was partially impaired (Fig. 10, centre columns of graphs in the SAME and DIFF blocks). The overall information about the position in space of the target trajectory shape was maintained, as well as a partial amount of information about the shape itself. In this case, the difference between the measured errors in the SAME and DIFF conditions was higher (Fig. 11). Indeed, a two-way ANOVA revealed a significant effect of the interaction between the presence of motor lesions (LESION) and the two conditions (TEST) (*TEST*$$F(1,393)=7.53$$, $$p=0.006$$; *LESION*$$F(1,393)=796.301$$, $$p<0.001$$; $$TEST\times LESION$$$$F(1,393)=18.7$$, $$p<0.001$$).

In the case of the FULL_LESION simulations, the resulting behaviour during the test phase was destructively altered (Fig. 10, right column). The overall information about the position in space of the target trajectory shape was still maintained in the SAME condition, but almost no information about the shape itself was retained. In the DIFF condition, no information was maintained at all, even information regarding the position in space of the target trajectory shape. In this case, the difference between the measured errors in the SAME and DIFF conditions became dramatic (Fig. 11). A two-way ANOVA revealed a significant effect of the interaction between the presence of motor lesions (LESION) and the two conditions (TEST) (*TEST*$$F(1,393)=2.71$$, $$p<0.001$$; *LESION*$$F(1,393)=7.03$$, $$p< 0.001$$; $$TEST\times LESION$$$$F(1,393)=10.872$$,$$p<0.001$$).

A video showing the SAME test conditions for the three simulation types is given in Online Resource 3.

## Discussion

The simulations presented here show that the disinhibitory process implemented by the basal ganglia can be a reliable mechanism for the selection of the internal dynamics within a single cortical module. In particular, the simulation in Sect. 5.1 shows that basal ganglia disinhibition is sufficient to select differential cortical dynamics. These dynamics can be read out to control the activity of motor actuators, exploiting the ability of dynamical reservoirs to learn sequences and generalize the relations between the input and output spaces, as shown by the ability to generalize motor trajectories over translation and scale.

The simulation in Sect. 5.1 also shows that the control of this selection can be suitably modulated by striatal dopamine. In the simulation, the dopaminergic efflux regulates the efficacy in the initiation, termination and lock-in of basal ganglia selection. As a result, during the test phase the cortical dynamics are switched to a “neutral” state when striatal dopamine level is low. When striatal dopamine level is set to a higher level, the class of cortical dynamics is determined by selection in the basal ganglia so that the correct read-out signals are produced. Such a role of dopamine in motor control is consistent with what is observed in Parkinsonian patients. Indeed, these patients show deficits in learning new motor abilities (Muslimovic et al. 2007; Abbruzzese et al. 2009; Espay et al. 2011), as well as in skilled movements such as handwriting and graphical tasks (Rosenblum et al. 2013; Tucha et al. 2006), due to a reduced efficacy of basal ganglia action caused by low levels of dopamine. Notably, micrographia, a peculiar handwriting deficit shown by Parkinsonian patients (McLennan et al. 1972; Kim et al. 2005; Jankovic 2008; Ma et al. 2013) has been qualitatively reproduced by the model described here when the external afferents to the striatal layers are partly or totally lesioned (second set of simulations, Fig. 10).

Simulations also show that basal ganglia disinhibition can play several roles. First, basal ganglia disinhibition potentiates how cortical modules sparsify the input in time and space. Sparsification in time and space is a fundamental property of reservoirs. Thanks to this property reservoir, networks allow a linear solution of problems that are originally nonlinearly separable. In the model, the striatum filters the coarse-grained information of the input. Based on this coarse information, basal ganglia disinhibition persistently enhances the dynamics of differential cortical sub-populations (Fig. 7). This focussed enhancement *amplifies the sparsification processes of the cortical module*. Based on this strengthened sparsification, the cortical module can learn multiple radically different mappings between input and output signals while limiting possible interference effects. The third set of simulations illustrates this enhancing effect of basal ganglia disinhibition. The reproduction error measured in the SAME test condition of the PARTIAL_LESION simulation is significantly higher than the error in the SAME test condition of the BASELINE simulation. This is a clear evidence that the primary motor cortical module without the disinhibition by the primary motor basal ganglia fails to sufficiently sparsify the input so as to suitably map it to the target movement trajectory. Instead, the motor cortex with the enhancement of the basal ganglia can learn all the target trajectories without interference in correspondence to the coarse information related to the different movements.

Second, basal ganglia disinhibition preserves coarse-grained information *throughout cortico-corticalpathways*. This property derives from the same enhancement effect discussed above. When a cortical subpopulation is enhanced a strong mark is impressed to the dynamics of the cortical module. This mark can be easily exploited by both cortico-cortical and cortico-striatal learning processes when information traverses multiple CSNTC modules. The third set of simulations shows the importance of this property. The reproduction error measured in the DIFF test condition of the FULL_LESION simulation is dramatically higher than the error in the other simulations, resulting in a completely disrupted behaviour. This dramatic effect is due to the impairment of the striatal activity of the high-level motor module that prevents coarse-grained information about the task to reach the primary motor cortex.

### Comparison with other models

The model presented here can be compared with the main computational hypotheses proposed in the literature to describe the functional interaction between cortex and basal ganglia by appealing to dynamical concepts as here. The models described by Wickens et al. (1994), Houk and Wise (1995), Beiser and Houk (1998) and Frank et al. (2001) share two common ideas. First, the activity of a cortical assembly or column is bistable, switching between a lower and a higher state. Second, the basal ganglia select which column to switch on through disinhibition based on a striatal internal competition. Wickens et al. (1994), starting from the Hebbian hypothesis of cell assemblies, proposed that the control of motor programs is implemented by the cortex through the ignition of cortical assemblies. In their model, when cells belonging to an assembly are activated over a threshold, a reaction chain leads to the ignition of all the other cells in the same assembly. This process ends with a stable activation of the whole assembly that is then sufficient to trigger a motor program. The role ascribed to the selection process of the basal ganglia is to differentially amplify the activation of cortical assemblies. In the authors’ hypothesis, as cortical assemblies reaching the striatum partially overlap, learning at the level of the cortico-striatal connections connects the activation of assemblies between each other, allowing the triggering of sequences of motor programs. Houk and Wise (1995) described a localistic firing-rate model of the interaction between thalamo-cortical loops and the basal ganglia in which the striatum acts as a context detector, linking motor behaviours to the right contextual patterns. Context is given by both the activity of the cortical column that is in loop with the striatal unit and by the activity of other cortical columns. Striatal functioning is based on a winner-take-all mechanism. The main feature of the model is that a temporary activation of a striatal unit produces a switch to a permanent higher activation of the target cortical column, which thus instantiates a memory of the context detected by the striatum. Beiser and Houk (1998) further investigated the computational hypothesis proposed by Houk and Wise (1995). They showed that a composition of such cortico-basal ganglia loops where cortico-striatal connectivity is randomly generated produces responses that are uniquely coupled to different sequences in the presentation of the cues. Frank et al. (2001) and O’Reilly and Frank (2006) described a computational model of working memory based on the prefrontal cortex and basal ganglia (the PBWM model) that is also built on the two principles described above. In the PBWM model, cortical modules are implemented as attractor networks whose dynamics are modified through an algorithm based on both Hebbian and error-driven learning. Basal ganglia selectively gate inputs to the cortical modules through disinhibition. When a channel is selected, the target cortical module receives external inputs that possibly drive the network to a new attractor state whereas when the channel is inhibited the previous attractor state is maintained. Contrary to the previous models, the cortical networks of the PBWM model can store and learn several attractor states. This feature, combined with the temporal selective gating of the basal ganglia, allows the model to solve complex working memory tasks. This model, as some of the others described above, is meant to describe working memory in the prefrontal cortex more than motor control in the motor cortical areas, although, as also noted by the authors, working memory and motor control rely on similar principles. This contiguity also emerges on the anatomical level, where the microstructure of the interaction between the prefrontal cortex and the ventro-medial basal ganglia is the same as that between the motor cortices and the dorsal basal ganglia. Notwithstanding their power, none of the four models above give a full account of the dynamic nature and integration of high-level motor control (selection, initiation, modulation and termination of movements) and the very implementation of the motor programs.

A different model was proposed by Dominey (1995) in which the first idea of a cortical dynamical reservoir (see Sect. 2.1) was proposed in the implementation of a prefrontal cortex module that controls the basal ganglia disinhibition to the superior colliculus for saccade generation. In this model the temporal dynamics of the cortical module allows the system to learn, through reinforcement learning, to control oculomotor behaviour. Differently from the model presented here, however, selection in the basal ganglia is not implemented and the re-entrant interaction between cortex and the basal ganglia is not reproduced.

The model presented here is coherent with the described works because it implements both context detection by the basal ganglia, here in a biological plausible way, and maintenance of cortical states through the release of thalamo-cortical loops. However, our model has also important novelties with respect to the described models. First, it merges the property of maintaining a memory of the current state (Wickens et al. 1994; Houk and Wise 1995; Beiser and Houk 1998; Frank et al. 2001), with the property of producing an internal dynamics in response to the input (Dominey 1995), so that the evidence of complex neural activity can be reconciled with the role of frontal cortex in working memory, in line with the duality of electrophysiological data in which both maintenance of activity patterns (Georgopoulos et al. 1982; Scott 2003) and complex temporal dynamics (Hatsopoulos et al. 2007; Afshar et al. 2011; Churchland et al. 2012) are found in the same cortical circuits. Second, it implements the interaction between dopamine-based basal ganglia selection and cortical activity in a plausible biological way. Thus our model explains in detail how motor programs are selected and performed.

## Conclusions

We described a model that proposes a hypothesis on the mechanisms of interaction between cortex and the basal ganglia. The model was built by integrating reservoir computing as a model of cortex, and cumulative competition leading to disinhibition as a model of the basal ganglia. The model shows that selection of the basal ganglia can control cortical activity by drastically changing its dynamics. In particular, selection substantially improves the sparsification processes within cortex. It also explains how cortical activity can transiently maintain information while producing complex temporal patterns of activation. This is made possible by the basal ganglia imposing specific dynamics to the selected cortical subpopulations.

Notwithstanding these strengths the model does not explain at least two important issues, representing two possible starting points for future work. First, all simulations presented here use supervised learning to update cortical read-out connections, so the model does not explain how such connections might be acquired with learning processes typical of cortex, in particular Hebbian learning (Arai et al. 2011; Koch et al. 2013) and possibly trial-and-error learning (Hoerzer et al. 2014). We restricted our implementation to supervised learning because of its large use in reservoir computing, and the consequent availability of algorithms to solve technical problems, given that our focus was on the system-level interaction between the basal ganglia and cortex and not on learning processes. Unsupervised and error-driven learning in reservoir computing are starting to be studied only recently (see Legenstein et al. 2009; Hoerzer et al. 2014 for an promising solution to reward-driven learning).

The second issue that the model does not face involves the effects of closed-loop interactions with the environment on learning. Realistic motor control operates within a closed-loop system involving the brain, the body and the environment where the motor acts exerted by the animal generate a continuous feedback from the environment. This feedback is readily integrated by the brain to control and modulate the motor acts themselves. For example, the activity of the primary motor cortex is continuously modulated by somatosensory information (here abstracted with a sinusoidal input), or by motor efferent copies, during movement performance. Modelling the effects of this modality of interaction with the environment is very important as it produces relevant effects on the nature of motor control and performance. Some solutions have been developed with the aim to manage online feedback in reservoirs networks while updating the read-out weights (Steil 2004; Sussillo and Abbott 2009). Simulating the model with a more realistic input will be a goal of future research.

An interesting issue that can be investigated with the model is the relation between learning equilibrium-points and learning complex motor trajectories. Are these two different possible modalities of motor learning or are they interrelated? Do they share the same neural substrates? Section 5 highlighted that the model, exploiting the properties of dynamical reservoirs, can learn to reach an equilibrium-point posture (Feldman 1986; Bizzi et al. 1992; Caligiore et al. 2014) alongside learning rhythmic complex trajectories. Furthermore, the learning of constant read-out activities should be easier to achieve by a reward-driven learning algorithm. Thus, we speculate that initial learning of a complex trajectory might be guided by a reward-based acquisition of few intermediate key points relevant for the whole motor trajectory. These via points might then scaffold the acquisition of the final accurate trajectory, in particular on the basis of cortico-cortical learning processes and possibly the contribution of cerebellum (Wolpert et al. 1998; Shadmehr and Krakauer 2008; Caligiore et al. 2013).

### Electronic supplementary material

Supplementary material 1 (mpg 8196 KB)

Supplementary material 2 (mpg 28118 KB)

Supplementary material 3 (mpg 15874 KB)

Supplementary material 4 (pdf 282 KB)

## Figures and Tables

**Fig. 12:**
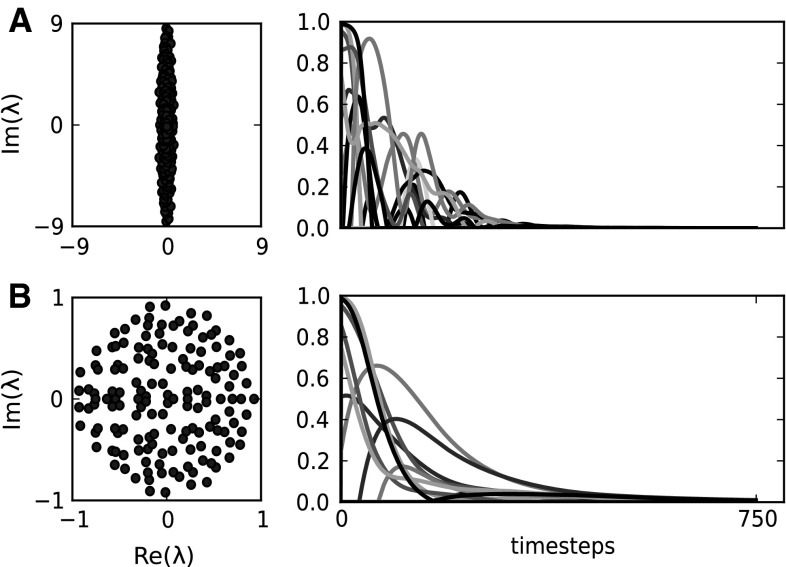
For each row of graphs, the *left graphs* represent the eigenvalues of the weight matrix. **a** An echo state network where the weight matrix is built with $\psi = 0.2$ and $\epsilon = 0.0001$. Notice that the high variability of its dynamics while fading. **b** A standard echo state network ($\psi = 0.5, \epsilon = 0.0001$)

## Appendix 1: Optimizing the weights of leaky-integrator reservoirs

Generating suitable connection weights of a reservoir network to optimize the learning of the read-out weights for a given task is not always easy. In particular, there is not a systematic way to modulate the temporal variability of the network response. The richness of the temporal response of a reservoir network to an input is the founding element of its computational expressiveness. Indeed, networks with richer dynamics can reproduce in their read-out units nonlinear temporal trajectories with an high definition. In other words, the more the states that the network visits in time are dissimilar from one another (low correlation between states), the more the output function that the network can learn to reproduce can be complex. Here we describe a heuristic algorithm that we have developed, and used in the models presented here, to improve the temporal variability of echo state reservoirs. In a dynamical system of type $\dot{{\mathbf {x}}} = {\mathbf {W}}{\mathbf {x}}$, the properties of the matrix ${\mathbf {W}}$ determine its temporal dynamics. In particular, the real and imaginary components of the eigenvalues of ${\mathbf {W}}$ determine, respectively, the amount of infinitesimal contraction/expansion or infinitesimal rotation in the phase space of the system (Stender 2007). Thus, a matrix with eigenvalues having high imaginary and low real components produces dynamics with high infinitesimal rotations and a low amount of contraction/expansion. Infinitesimal rotations in the dynamics correspond to low correlation between successive states; that is, richer dynamics. It is possible to build a matrix with these properties starting from a random square matrix ${\mathbf {M}}\in \mathfrak {R}^{n\times n}$. This matrix ${\mathbf {M}}$ is equivalent to the sum of two matrices ${\mathbf {M}}_\mathrm{sim}+{\mathbf {M}}_\mathrm{skew}$, where ${\mathbf {M}}_\mathrm{sim}=\left( {\mathbf {M+M^T}}\right) /2$ is a symmetric matrix with all nonzero eigenvalues being pure real and ${\mathbf {M}}_\mathrm{sim}=\left( {\mathbf {M-M^T}}\right) /2$ is a skew-symmetric matrix with all nonzero eigenvalues being pure imaginary. Given this property, we can build a matrix ${\mathbf {M}}_\mathrm{rot}=\psi \cdot {\mathbf {M}}_\mathrm{sim}+(1-\psi )\cdot {\mathbf {M}}_\mathrm{skew}$, where the parameter $\psi$ is the proportion between the amplitude of real versus imaginary eigenvalues of ${\mathbf {M}}_\mathrm{rot}$. By setting a small value of $\psi$ (in particular $0<\psi <0.5$) one can obtain a matrix that determines an higher amount of rotation and a lower amount of contraction/expansion than the original matrix. We can now use this matrix to built the matrix of weights of an echo state network of *n* units by normalizing it to a matrix $\mathbf {M}_{norm}$ so that $\left( 1-\epsilon \right) < \rho \left( \left( \delta t/ \tau \right) \cdot \mathbf {M}_{norm} + \left( 1 - \left( \delta t/\tau \right) \right) \cdot I \right) < 1$, where $\rho (.)$ is the spectral radius (Jaeger et al. 2007). Figure 12 shows how a reservoir with such a modification has a richer response to an impulse during its fading dynamics.

## Appendix 2: Parameters of the simulations

### Numerical integration of the units of the model

**Table:**

*Basal ganglia units*
$$dt =\mathtt{0.001 }$$
$${\tau } =\mathtt{0.005 }$$
$${th} =\mathtt{0.0 }$$
$${\alpha } =\mathtt{1.0 }$$
$${bl_{D1}} =\mathtt{0.1 }$$
$${bl_{D2}} =\mathtt{0.2 }$$
$${da_{D1}} =\mathtt{0.5 }$$
$${da_{D2}} =\mathtt{5.0 }$$
$$\hbox {Channels} =\mathtt{3 }$$
*Thalamic units*
$${dt} =\mathtt{0.001 }$$
$${\tau } =\mathtt{0.005 }$$
$${th} =\mathtt{0.0 }$$
$${\alpha } =\mathtt{1.0 }$$
$$\hbox {n} =\mathtt{3 }$$
*Cortical units*
$${dt} =\mathtt{0.001 }$$
$${\tau } =\mathtt{0.005 }$$
$${th} =\mathtt{0.0 }$$
$${\alpha } =\mathtt{1.0 }$$
$$n =\mathtt{300 }$$
$$\hbox {Sparseness} =\mathtt{1.0 }$$
$${\epsilon ^*} =\mathtt{0.0001 }$$

**Weights**

*Intrinsic connections of the basal ganglia*

**Table:**

To	From			
	GPe	STN	$\hbox {s}_{\mathrm{D1}}$	$\hbox {s}_{\mathrm{D2}}$
GPi	$\mathtt{-0.1 }$	3.0	$\mathtt{-3.0 }$	.
GPe	.	2.0	.	$\mathtt{-2.5 }$
STN	$\mathtt{-1.5 }$	.	.	.

*Connections between the components of the CSNTC module*

**Table:**

To	From			
	GPi	Tha	c	Others
$\hbox {s}_{\mathrm{D1}}$	.	.	0.3	0.8
$\hbox {s}_{\mathrm{D2}}$	.	.	0.25	0.8
STN	.	.	1.0	.
Tha	$-$ 30.0	.	0.4	.
c	.	0.6	.	.

**Learning**

*Offline learning*

**Table:**

Read-out weights—regression	
$${\lambda ^2} =\mathtt{0.000001 }$$	

*Online learning*

**Table:**

Read-out weights—BPDC	
$${\beta } =\mathtt{0.00001 }$$	
$${\eta } =\mathtt{0.2 }$$	
Cortico-striatal weights—Oja’s rule	
$${\eta _{sc}} =\mathtt{0.05 }$$	
$${k_{WTA_{Str}}} =\mathtt{1}$$	
$${k_{WTA_{Ctx}}} =\mathtt{30 }$$	

## Footnotes

1 These studies also focus on the idea that learning to select sequences of channels allows the acquisition of complex motor actions.
