# Supplementary material for: Selection of cortical dynamics for motor behaviour by the basal ganglia
Source: Biol Cybern. 2015 Nov 4;109(6):575–95. doi: 10.1007/s00422-015-0662-6 (PMC4656718; doi:10.1007/s00422-015-0662-6)
Supplement: Supplementary file 4 — Supplementary material 4 (pdf 282 KB) [file 422_2015_662_MOESM4_ESM.pdf]

Biological Cybernetics manuscript No.
(will be inserted by the editor)

Supplementary material to "Selection of Cortical Dynamics for Motor Behaviour by the Basal Ganglia"

Francesco Mannella · Gianluca Baldassarre

Received: date / Revised: date

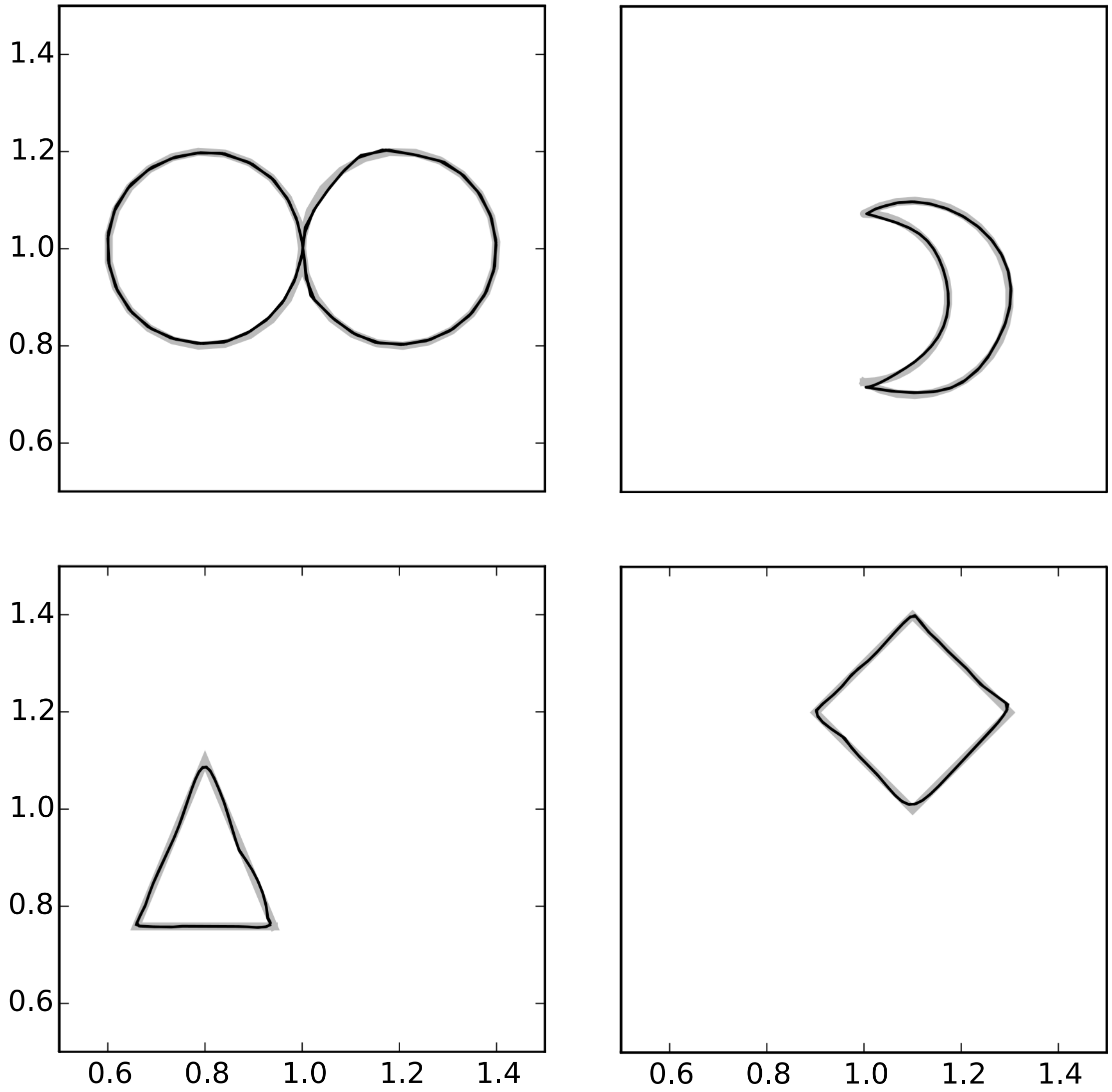

Fig. 1 Performance of the model composed of a single cortico-striato-nigro-thalamo-cortical loop (LOOP_MODEL in the main paper): selection between four different trajectories. Each graph shows the behaviour of the controlled 2D arm in case of the selection of one of the four basal ganglia channels based on the information about the task. Bold light gray curves denote the target trajectories that were to be learned. The thinner curves show the trajectories effectively performed in the four target tests.

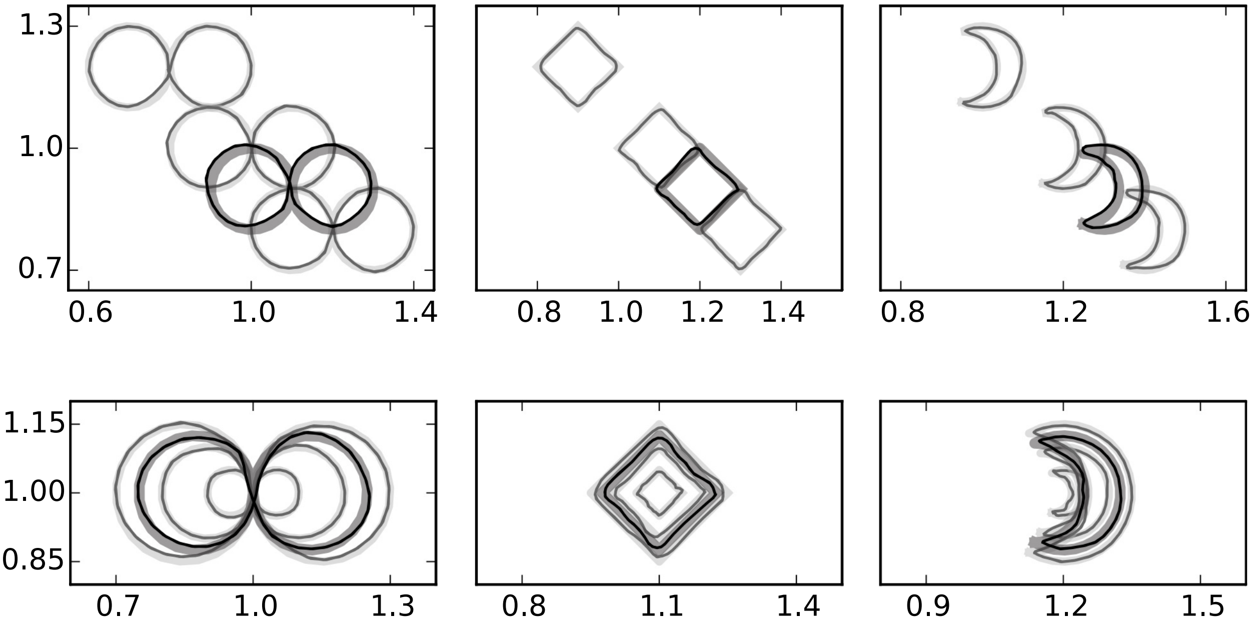

Fig. 2 Performance of the model composed of a cortico-striato-nigro-thalamo-cortical loop (LOOP_MODEL in the main paper): generalization over scaling and translation with complex trajectories. Each column of graphs shows the behaviour of the controlled 2D arm in case of the selection of one of the three basal ganglia channels based on the information about the task and the sensory context. Bold light gray curves denote the target trajectories that were to be learned. Bold dark gray curves denote the trajectories expected during the generalization tests. The thinner curves show the trajectories effectively performed in the three target tests and in the generalization tests. The top row of graphs shows the case in which the same trajectory has been learned in three different spatial positions. The bottom row shows the case in which the same trajectory has been learned at three different scales.
